# Optogenetic Probing and Manipulation of the Calyx-Type Presynaptic Terminal in the Embryonic Chick Ciliary Ganglion

**DOI:** 10.1371/journal.pone.0059179

**Published:** 2013-03-21

**Authors:** Ryo Egawa, Shoko Hososhima, Xubin Hou, Hidetaka Katow, Toru Ishizuka, Harukazu Nakamura, Hiromu Yawo

**Affiliations:** 1 Department of Developmental Biology and Neuroscience, Tohoku University Graduate School of Life Sciences, Sendai, Japan; 2 Japan Science and Technology Agency (JST), Core Research of Evolutional Science & Technology (CREST), Tokyo, Japan; 3 Tohoku University Institute for International Advanced Research and Education, Sendai, Japan; 4 Department of Molecular Neurobiology, Institute of Development, Aging and Cancer, Tohoku University, Sendai, Japan; 5 Center for Neuroscience, Tohoku University Graduate School of Medicine, Sendai, Japan; University of Houston, United States of America

## Abstract

The calyx-type synapse of chick ciliary ganglion (CG) has been intensively studied for decades as a model system for the synaptic development, morphology and physiology. Despite recent advances in optogenetics probing and/or manipulation of the elementary steps of the transmitter release such as membrane depolarization and Ca^2+^ elevation, the current gene-manipulating methods are not suitable for targeting specifically the calyx-type presynaptic terminals. Here, we evaluated a method for manipulating the molecular and functional organization of the presynaptic terminals of this model synapse. We transfected progenitors of the Edinger-Westphal (EW) nucleus neurons with an EGFP expression vector by *in ovo* electroporation at embryonic day 2 (E2) and examined the CG at E8–14. We found that dozens of the calyx-type presynaptic terminals and axons were selectively labeled with EGFP fluorescence. When a Brainbow construct containing the membrane-tethered fluorescent proteins m-CFP, m-YFP and m-RFP, was introduced together with a Cre expression construct, the color coding of each presynaptic axon facilitated discrimination among inter-tangled projections, particularly during the developmental re-organization period of synaptic connections. With the simultaneous expression of one of the chimeric variants of channelrhodopsins, channelrhodopsin-fast receiver (ChRFR), and R-GECO1, a red-shifted fluorescent Ca^2+^-sensor, the Ca^2+^ elevation was optically measured under direct photostimulation of the presynaptic terminal. Although this optically evoked Ca^2+^ elevation was mostly dependent on the action potential, a significant component remained even in the absence of extracellular Ca^2+^. It is suggested that the photo-activation of ChRFR facilitated the release of Ca^2+^ from intracellular Ca^2+^ stores directly or indirectly. The above system, by facilitating the molecular study of the calyx-type presynaptic terminal, would provide an experimental platform for unveiling the molecular mechanisms underlying the morphology, physiology and development of synapses.

## Introduction

It is a generally accepted idea that transmitter release from the presynaptic terminal consists of several elementary processes [1]–[6]. Upon invasion of an action potential into a presynaptic terminal, the voltage-dependent Ca^2+^ channels (VDCCs) are activated by brief depolarization. This is followed by Ca^2+^ inflow into the nerve terminal according to the electrochemical gradient. The consequent focal elevation of the intracellular Ca^2+^ concentration triggers a molecular cascade that causes the exocytosis of neurotransmitter in the synaptic vesicles. These vesicles are retrieved by endocytosis, refilled and recycled for the subsequent transmitter release. Each of these elementary processes is also the principal target of short- and long-term modulation of the transmitter release.

As one of the model systems of neuro-neuronal synapses, the calyx-type large synapses that are formed in the ciliary ganglion (CG), a parasympathetic ganglion behind the eye, of chick embryo have been intensively studied morphophysiologically for decades. The neurons in the Edinger-Westphal (EW) nucleus in the midbrain innervate the ganglion through the oculomotor nerve and form functional cholinergic synapses on the ciliary cells between stage 26 (E5) and 33 (E8) [7]–[9]. In these early stages the postsynaptic ciliary cells have dendrites with multiple, small boutons [7]. By stage 36 (E10)–40 (E14), a single presynaptic terminal expands to form a calyx which engulfs a large part of the postsynaptic cell soma [7], [10]–[12]. Physiologically, this calyx-type synapse has been a model of one-to-one-type neuronal synapses for the investigation of presynaptic mechanisms [13]–[15]. With its relatively large size, the chick CG was the first vertebrate preparation in which one can measure the membrane potential and the membrane current directly from a presynaptic terminal [16]–[19]. It was also the first vertebrate synapse in which Ca^2+^-dependent dye fluorescence was measured from a single presynaptic terminal [20].

Recently, various genetically-encoded tools have come to the forefront of neuroscience that show promise for presynaptic studies. For instance, if the Brainbow technique, which allows color coding of the inter-tangled morphology [21], [22], could be applied to the calyx-type synapse, it should facilitate the analysis of axonal branching and synapse formation. Elucidating the physiology of presynaptic terminals is expected to be facilitated by the recently developed optogenetics that probe the elementary steps of transmitter release such as Ca^2+^ elevation and exo/endocytosis [23] and/or allow artificial manipulation of the presynaptic functions [24], [25]. However, it has been difficult using the current gene-manipulating methods to target specifically such peculiar presynaptic terminals.

In this study, we evaluated the effectiveness of gene manipulation in the chick CG synapse using an *in ovo* electroporation technique [26], [27]. We found that this technique could be a useful tool for inducing various genetic modifications in presynaptic neurons. Consequently, this classical model synapse can be revived as a novel experimental platform for studying the morphology, physiology and development of the presynaptic terminal.

## Results

### Evaluation of Multiple Gene Expressions in the Calyx-type Presynaptic Terminal

**Figure 1:**
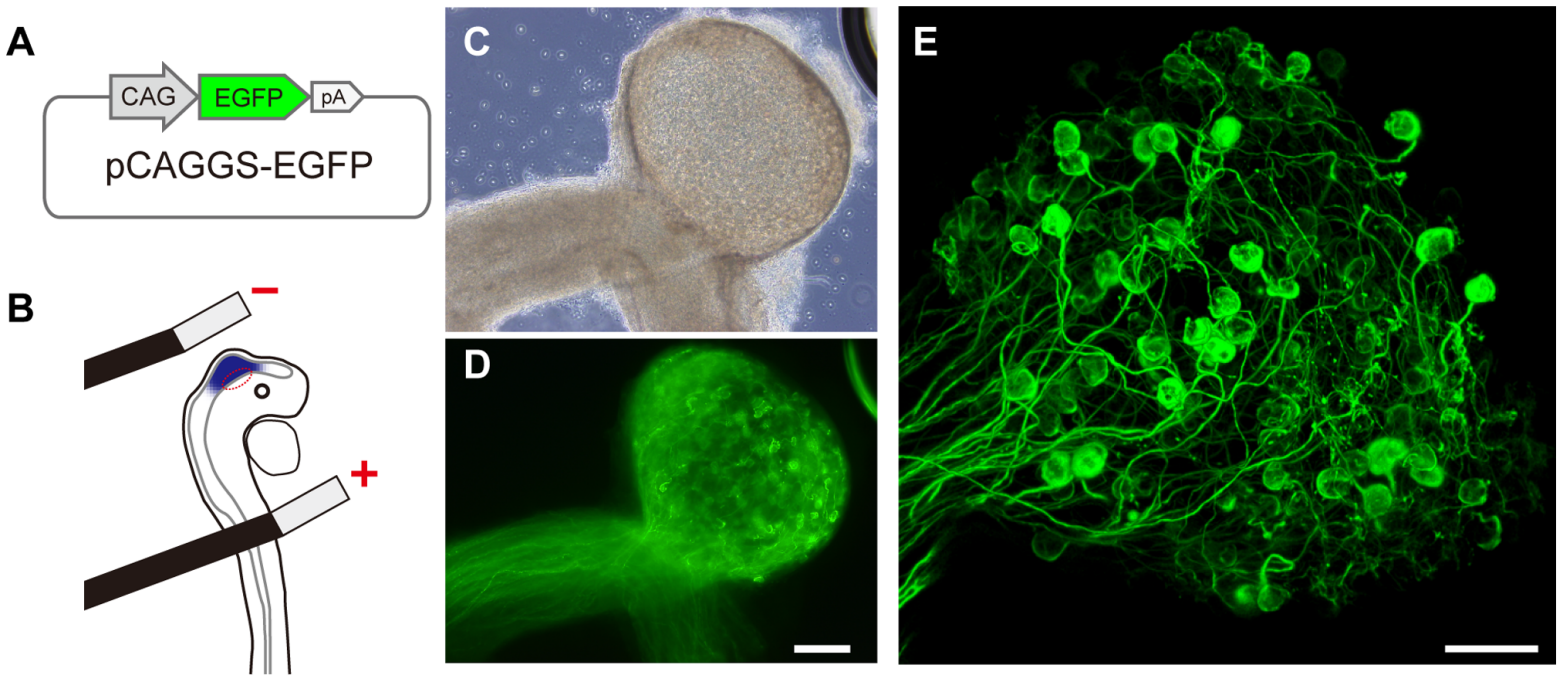
Genetic manipulation of presynaptic neurons innervating chick ciliary ganglion using *in ovo* electroporation method. **A**, Schematic structure of pCAGGS-EGFP plasmid vector. **B**, Position of bipolar electrodes placed on the midbrain region (red ellipse) of E2 embryo. **C** and **D**, Bright-field and EGFP fluorescent images of isolated CG with oculomotor nerve of E14 embryo. **E**, A compiled image of a CG (E14) under confocal microscopy. Scale bars: 200 µm for **D** and 100 µm for **E**.

As one of the methods to introduce genes of interest into the chick CG presynaptic terminal, we adopted an *in ovo* electroporation technique [26], [27]. We injected pCAGGS-EGFP into the E2 midbrain, where the CG presynaptic neurons are generated, and served to electroporation (Fig. 1A and 1B). At E4, EGFP fluorescence was observed in the midbrain region and in the oculomotor nerve extending from it. At E14, EGFP fluorescence was detectable in the neurons in the EW nucleus, which innervate the CG through the oculomotor nerve, the axons in the oculomotor nerve and the large calyx-type presynaptic terminals (Fig. 1C–1E and Video S1). Fluorescence was negligible in the Schwann cells and the postsynaptic ciliary and choroidal cells. Thus, in CG, transgene expression was localized only in the presynaptic neuron.

**Figure 2:**
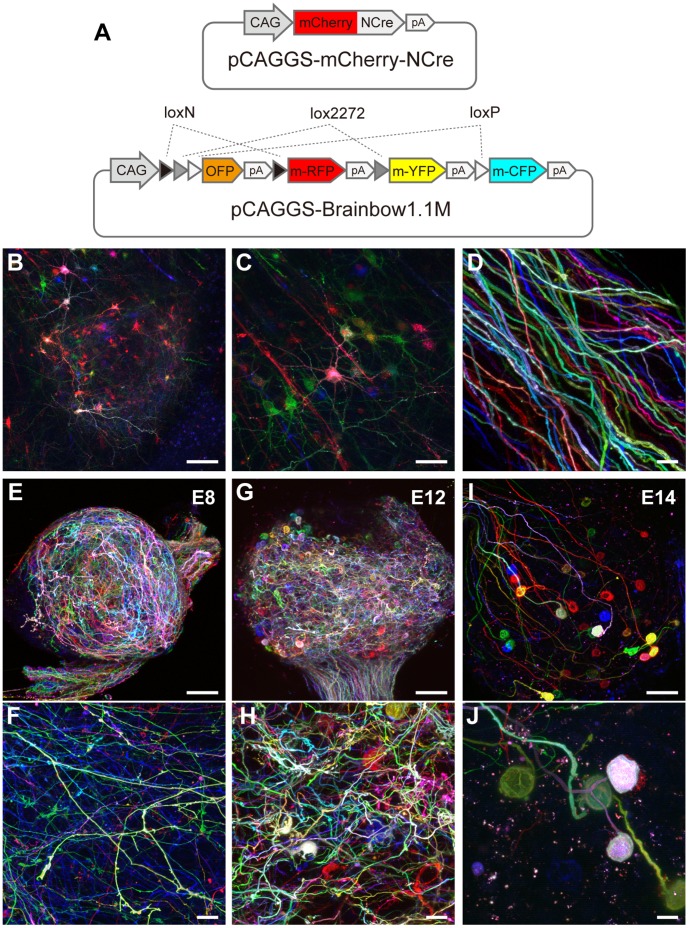
Mosaic expression of fluorescent proteins with Brainbow strategy. A , Schematic structures of injected plasmid vectors, pCAGGS-mCherry-NCre (top) and pCAGGS-Brainbow1.1M (bottom). **B**, Compiled image of a sagittal section of the midbrain (E14) under confocal microscopy. The neurons, which are colored according to the combination of expressed m-XFPs, are clustered in the EW nucleus (center). **C**, An enlarged image of the EW nucleus. Note that each neuron expresses mCherry in the nucleus (arrows). **D**, Oculomotor axons (E14). **E** and **F**, a CG from E8 embryo. **G** and **H**, other CG from E10 embryo. **I** and **J**, other CG from E14 embryo. Arrows indicate debris of the axonal membrane. Scale bars: 100 µm for **B**, **E**, **G** and **I**; 50 µm for **C** and 20 µm for **D**, **F**, **H** and **J**.

For the further identification of inter-tangled axons and their terminal endings, we speculated that application of the Brainbow technique, by which individual neurons and axons are currently labeled with various combinations of fluorescent markers [21], would be promising. To test the effectiveness of the Brainbow technique, we electroporated a mixture of two plasmid vectors, pCAGGS-Brainbow1.1M, which contains three membrane-tethered fluorescent proteins (m-XFPs), m-CFP, m-YFP and m-RFP, spaced by the sequences of loxP and its variants (Fig. 2A), and pCAGGS-mCherry-NCre in the E2 midbrain. Since co-electroporated plasmids are introduced in the same neuroblast, it is well expected that the cDNAs of the three membrane-tethered fluorescent proteins (m-XFPs) would be expressed at various combinations as a result of the stochastic Cre/loxP recombination of the Brainbow1.1M cassette. Indeed, in the EW nucleus at E14, each neuron expressed unique combinations of m-XFPs in the membrane and mCherry fluorescence in the nucleus (Fig. 2B and 2C). Individual axons running in the oculomotor nerve were also traceable for various lengths as they were distinctly labeled with the combination of m-XFPs (Fig. 2D).

Next, we traced axons and their terminal endings in the CG at various developmental stages. In the CG at E8, the axons were frequently branched and formed bouton-like but not calyx-type endings (Fig. 2E, 2F and Video S2). Some of the intraganglionic nerve endings acquired calyx-type configurations that covered the postsynaptic cells as early as at E10 (Fig. 2G, 2H and Video S3). Almost all axons formed calyx-type configurations in a one-to-one manner by E14 (Fig. 2I, 2J and Video S4). Debris of the axonal membrane, which was often a mixture of m-XFP, was abundantly formed around the calyx-type endings at stages E12–14.

### Optogenetic Probing and Manipulation

Recently, optogenetics has become a powerful approach to revealing the physiological properties of neurons and networks [28], either as sensors/probes to survey the cellular function, or as actuators/attenuators to up/down-regulate the neuronal activity. We tested R-GECO1 as sensors/probes for the presynaptic physiology, and channelrhodopsin-fast receiver (ChRFR) as the optogenetic actuator. R-GECO1 is a red-shifted fluorescent Ca^2+^-sensor with peak excitation at 560–580 nm [29]. ChRFR is one of the chimeric channelrhodopsins with improved expression in the cell membrane, reduced desensitization, and the fastest on-off photocurrent kinetics [30], [31].

**Figure 3:**
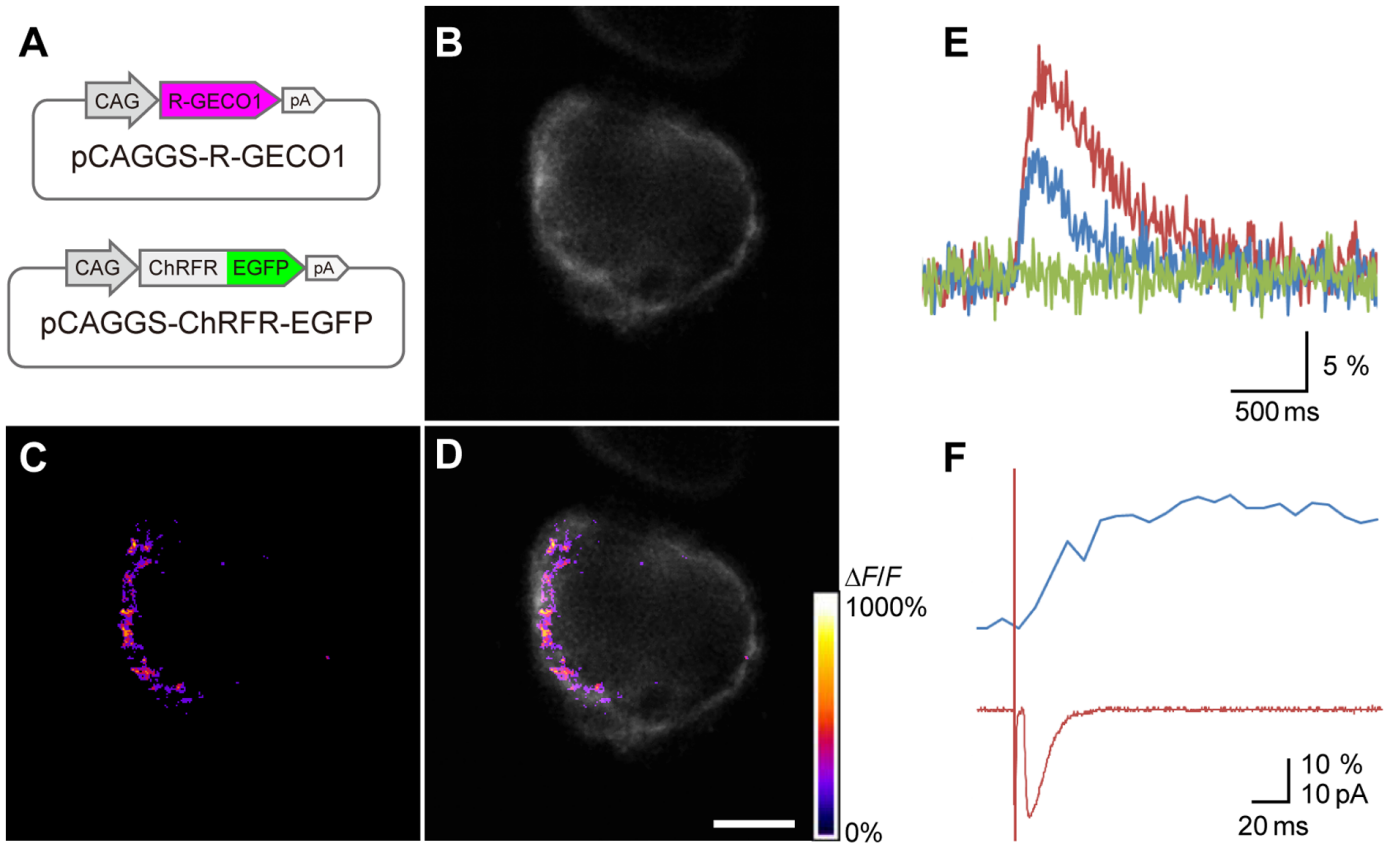
Ca^2+^ imaging of the calyx-type presynaptic terminal. A , Schematic structures of injected plasmid vectors, pCAGGS-ChRFR-EGFP (top) and pCAGGS-R-GECO1 (bottom). **B**, A confocal EGFP image of a calyx-type presynaptic terminal (E14) (optical slicing at 1.99 µm). **C**, A color-rated image of the *ΔF*/*F* of R-GECO1 immediately after electrical stimulation of the oculomotor nerve in the same optical slice. **D**, Overlay of B and C. Note that several hotspots are present in the synaptic face of the calyx. Scale bar, 10 µm. **E**, Time-dependent plots of bulky magnitudes of *ΔF/F* ([*ΔF/F*]_B_). The concentration of the extracellular Ca^2+^ was 5 mM (red), 2.5 mM (blue) and 0 mM (green). The oculomotor nerve was electrically stimulated as indicated (arrow). **F**, Simultaneous recordings of the [*ΔF/F*]_B_ (blue) and the EPSC (red).

The mixture of pCAGGS-R-GECO1 and pCAGGS-ChRFR-EGFP plasmid vectors was introduced in the E2 midbrain and the CG was examined at E14 (Fig. 3A and 3B). Although the R-GECO1 fluorescence was hardly detectable at rest, its increase in the presynaptic terminal was obvious in response to a single electrical stimulation of the oculomotor nerve (Video S5). Under close inspection, the *ΔF/F* of R-GECO1 was not uniformly elevated and several hot spots were detectable (Fig. 3C). The bulky magnitude of *ΔF/F* ([*ΔF/F*]_B_), where *F* is the average fluorescence in a calyx, was dependent on the extracellular concentration of Ca^2+^, and was negligible in its absence (Fig. 3D). Next, the EPSC was recorded simultaneously from the postsynaptic ciliary neuron under R-GECO1 imaging. As shown in Fig. 3E, the EPSC was evoked during the early rising phase of the presynaptic [*ΔF/F*]_B_.

**Figure 4:**
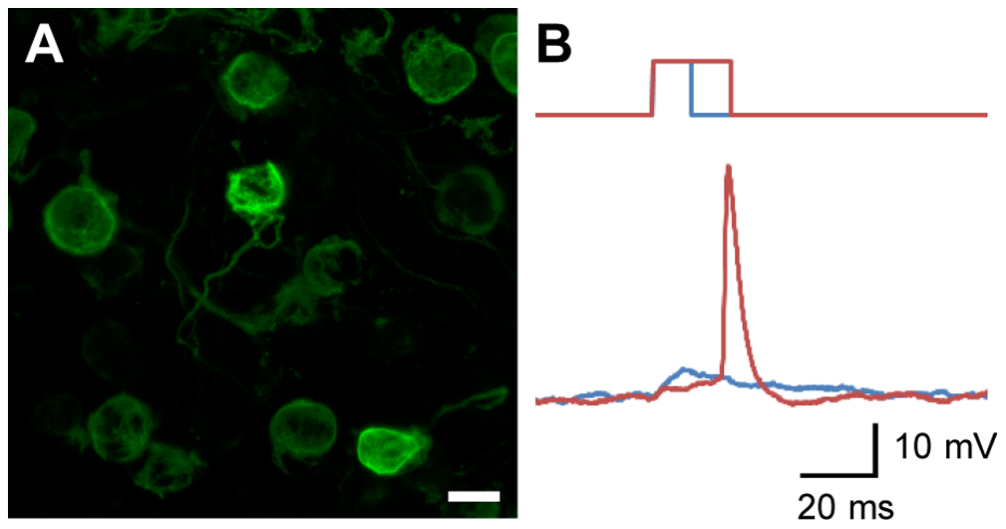
Direct optogenetic stimulation of calyx-type presynaptic terminals. A , Calyx-type presynaptic terminals expressing ChRFR-EGFP. Scale bar, 20 µm. **B**, Direct photostimulation with laser pulses of 10 ms (blue) and 20 ms (red) in the presence of 4-AP (1 mM). The resting potential, −53 mV; the action potential, 43 mV; the input resistance, 74 MΩ.

The ChRFR-EGFP-expressing calyx-type terminals were identified by their fluorescence at E14 (Fig. 4A). It was expected that the ChR-expressing nerve terminals would be directly stimulated by light. To test this, we put an optic fiber near the calyx-type presynaptic terminal and irradiated a blue laser flash (451 nm) directly on it while measuring the membrane potential of the same presynaptic terminal under current clamp. However, the light-evoked depolarization was frequently lower than the threshold depolarization required to evoke action potentials even at the maximal laser irradiance. This difficulty was somewhat overcome by the inclusion of 4-aminopyridine (4-AP), which blocks certain types of K^+^ channels in the perfusion solution (Fig. 4B). In this typical experiment, a relatively long pulse (duration ≥15 ms) was necessary to evoke an action potential at the maximal laser irradiance. The minimal pulse duration for evoking action potentials was 19±9.0 ms (range, 5–45 ms, n = 4).

**Figure 5:**
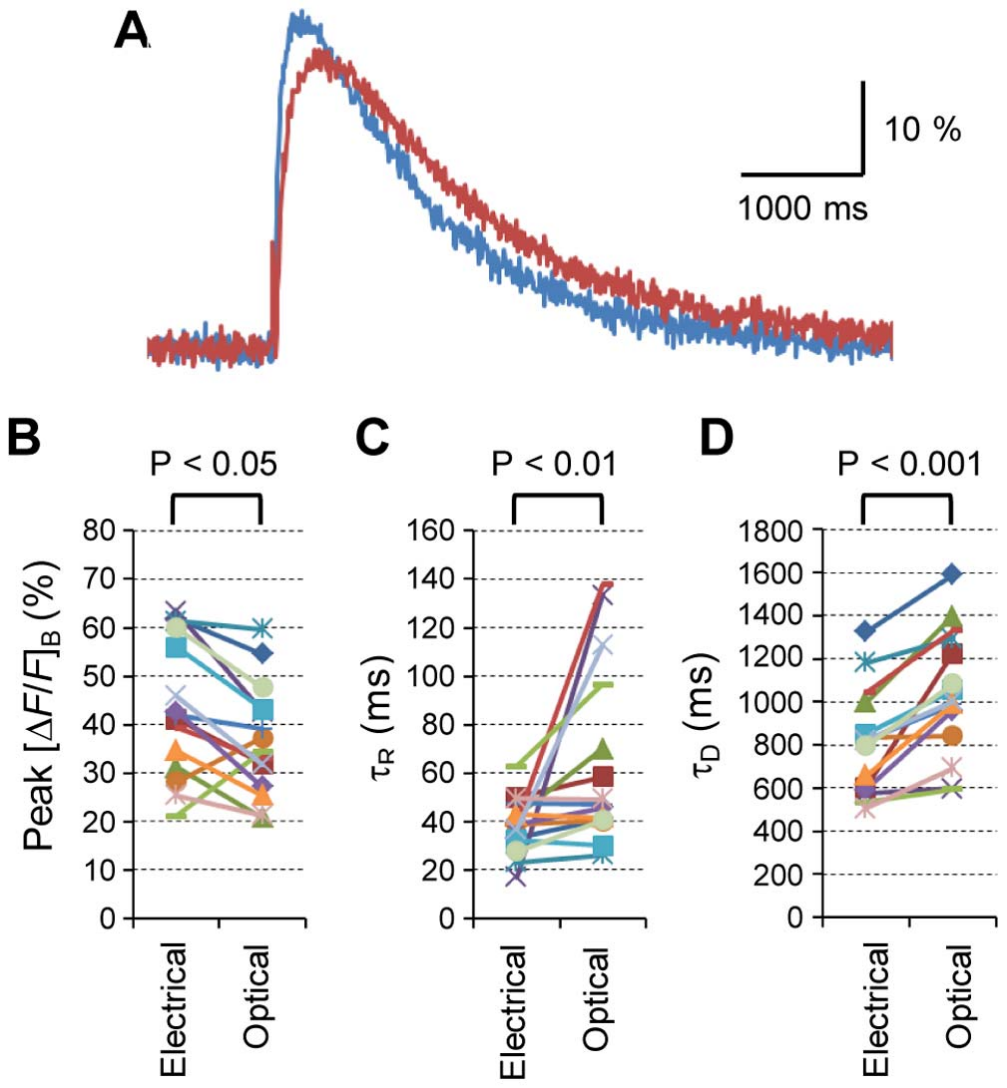
The intracellular Ca^2+^ transient induced by direct optogenetic stimulation of the presynaptic terminal. A , Sample [*ΔF/F*]_B_ of R-GECO1 responses evoked either by electrical stimulation of the oculomotor nerve (blue) or by direct photostimulation of the presynaptic terminal (red). The same calyx-type presynaptic terminal in the presence of 4-AP (1 mM). **B**–**D**, Quantitative comparison of Ca^2+^ transients between electrical stimulation and optogenetic stimulation: the peak [*ΔF/F*]_B_ (**B**), time constant of the rising phase (τ_R_, **C**) and that of the decaying phase (τ_D_, **D**). Each symbol indicates an individual presynaptic terminal.

Next, the [*ΔF/F*]_B_ of R-GECO1 was measured in response to the blue laser flash (20 ms) as well as conventional electrical stimulation of the oculomotor nerve in the presence of 4-AP. In the typical experiment shown in Fig. 5A, the [*ΔF/F*]_B_ was increased after single electrical stimulation of the axon, peaked in 200 ms and decreased with a time constant of 535 ms. On the other hand, the [*ΔF/F*]_B_ evoked by the laser flash was slightly lower in magnitude and slower in its time course with a time-to-peak of 400 ms and decaying time constant of 595 ms. As summarized in Fig. 5B, the peak [*ΔF/F*]_B_ was 44±3.6% by the electrical stimulation and 37±2.9% by the optical stimulation (n = 15, P<0.05). The time constant of the rising phase (τ_R_) was 39±3.0 ms by the electrical stimulation and 65±9.6 ms by the optical stimulation (n = 15, P<0.01) (Fig. 5C). The time constant of the decaying phase (τ_D_) was 810±63 ms by the electrical stimulation and 1040±75 ms by the optical stimulation (n = 15, P<0.001) (Fig. 5D).

**Figure 6:**
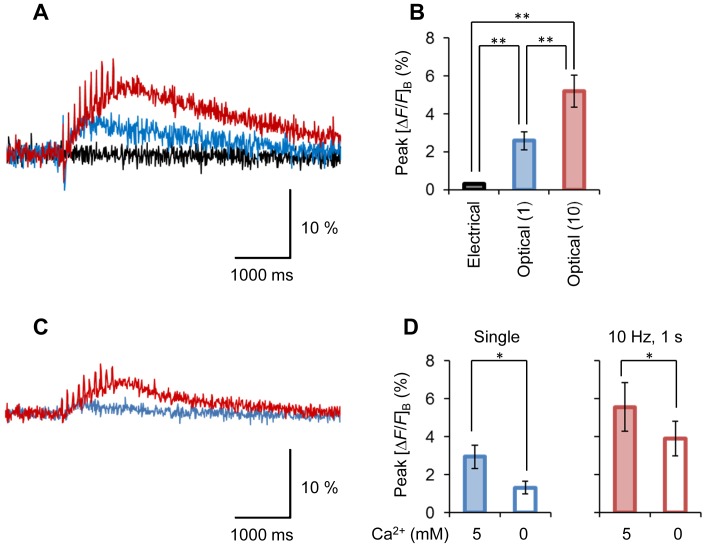
Optogenetic Ca^2+^ mobilization. **A**, Typical [*ΔF/F*]_B_ changes in the TTX-treated presynaptic terminal: the response to a single 20 ms laser pulse (blue), the response to a train of laser pulses (10 Hz) for 1 s (red) and the response to electrical stimulation (10 Hz, 1 s) to the oculomotor nerve (black). Each trace is an average of five consecutive records. **B**, Summary of peak [*ΔF/F*]_B_ changes (mean ± SEM) in the presence of TTX. Each column indicates (from left to right) the response to the train of electrical stimulations (10 Hz, 1 s), the single optical stimulation and the train of optical stimulations (10 Hz, 1 s). **, P<0.01 (n = 8). **C**, Sample [*ΔF/F*]_B_ responses of the same presynaptic terminal as shown in **A**, but with the extracellular Ca^2+^ being removed (EGTA, 1 mM). Each trace is an average of five consecutive records. **D**, The dependence of TTX-resistant [*ΔF/F*]_B_ changes (mean ± SEM) on the extracellular Ca^2+^ of 5 mM (left) and 0 mM (right): the response to single optical stimulation (left) and the response to a train of electrical stimulations (10 Hz, 1 s) (right). *P<0.05, two-tailed *t*-test (n = 5).

**Figure 7:**
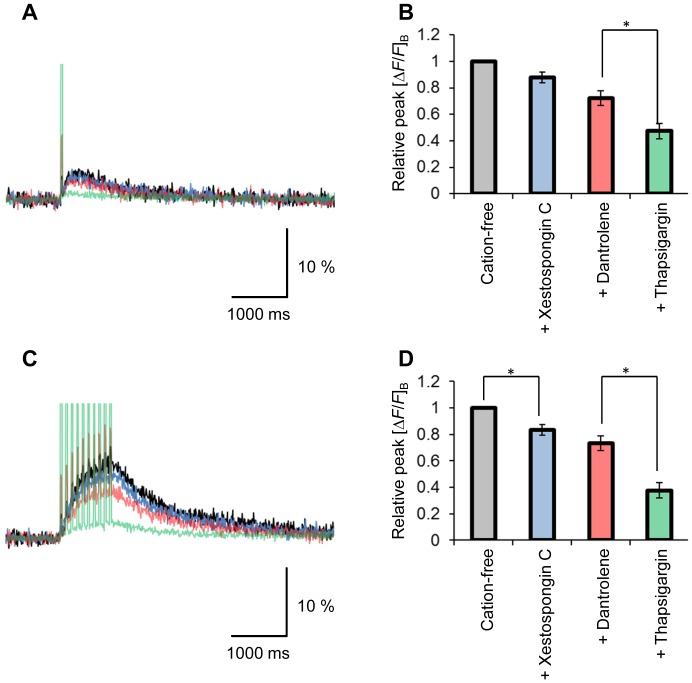
Involvement of Ca^2+^ store. **A**, Typical [*ΔF/F*]_B_ response of a calyx to a single 20 ms laser pulse in the cation-free extracellular solution (black), the response with additional xestospongin C (blue), the response with additional dantrolene (red) and the response after repetitive photostimulation with additional thapsigargin (green). Each trace is an average of five consecutive records. **B**, Summary of peak [*ΔF/F*]_B_ changes (mean ± SEM) in the cation-free solution. Each column indicates the relative value to that without any pharmacological reagents. *, P<0.05 (n = 7). **C**, Sample [*ΔF/F*]_B_ responses of the same presynaptic terminal as shown in **A**, but in response to a train of electrical stimulations (10 Hz, 1 s); without any pharmacological reagents in cation-free solution (black), with additional xestospongin C (blue), with additional dantrolene (red) and after repetitive photostimulation with additional thapsigargin (green). Each trace is an average of five consecutive records. **D**, Summary of peak [*ΔF/F*]_B_ responses to a train of electrical stimulations (10 Hz, 1 s) (mean ± SEM) in the cation-free solution. Each column indicates the relative value to that without any pharmacological reagents. *, P<0.05 (n = 7). Note in A and C that the artifactual fluorescence was increased during photostimulation after treatment with dantrolene, which emits green-yellow fluorescence [72].

Even in the presence of TTX, a substantial [*ΔF/F*]_B_ increase remained (Fig. 6A and 6B) with the direct photostimulation of the presynaptic terminal. The [*ΔF/F*]_B_ change was also evident when the stimulation was repetitively applied at 10 Hz for 1 s; however, it was negligible with electrical stimulation of the oculomotor nerve. Although the TTX-resistant [*ΔF/F*]_B_ increase was dependent on the extracellular Ca^2+^ concentration, a significant [*ΔF/F*]_B_ change was observed in a Ca^2+^-free condition with 1 mM EGTA (Fig. 6C and 6D). The [*ΔF/F*]_B_ increase in a Ca^2+^ free condition was 1.3±0.34% (a single photostimulation) and 3.9±0.92% (a train of 10 Hz for 1 s) with significant differences in the response to electrical stimulation (0.33±0.014%) (P<0.05, n = 5, two-tailed *t*-test). A substantial [*ΔF/F*]_B_ increase also remained in a cation-free extracellular solution containing 1 mM EGTA (Fig. 7A and 7B). It was slightly inhibited by 5 µM xestospongin C, a blocker of IP_3_ receptors, and 50 µM dantrolene, a non-selective blocker of ryanodine receptors, but the effects were insignificant. On the other hand, the [*ΔF/F*]_B_ decreased significantly when repetitively photostimulated in the presence of 2 µM thapsigargin. Similar pharmacological effects were observed for the [*ΔF/F*]_B_ evoked by repetitive photostimulation (a train of 10 Hz for 1 s) (Fig. 7C and 7D). However, the effects of xestrospongin C were significant.

## Discussion

This paper demonstrated that genetic modification of the chick midbrain neurons enabled the application of various optical methods for investigating the morphology and physiology of the presynaptic terminal. In this study, two kinds of plasmid vectors were frequently incorporated in the same midbrain neuron. This enabled us to label each neuron, its axonal branches and endings, with a unique combination of fluorescent markers when pCAGGS-Brainbow1.1M was incorporated with pCAGSS-NCre-mCherry. When ChRFR and R-GECO1 were expressed in the same presynaptic terminal, the presynaptic Ca^2+^ change in a single presynaptic terminal could be optically measured in response to a single flash of blue laser.

### Evaluation of Brainbow Expression Pattern

In both central and peripheral nervous systems, neurons have complex morphology with branching processes such as axons and dendrites. As many neurons are packed in a small space, it is often difficult to identify individual neurons or their processes. This problem has been solved using Brainbow strategies with which individual neurons can be induced to express different sets of fluorescent proteins in a mosaic pattern [21], [32]. In principle, the basic DNA constructs (Brainbow cassettes) consist of three of four genes encoding fluorescent proteins of different colors (XFPs) connected by loxP sites. With the aid of Cre-mediated DNA excision or inversion, the fluorescent proteins are stochastically expressed in various combinations. The individual neurons and their axons have thus been traced in transgenic systems such as mouse [21], *Drosophila* [33], [34] and zebrafish [35]. Otherwise, the mosaic expression of fluorescent proteins was attained using viral vectors encoding the Brainbow cassette [36]–[38].

Although the dendrites, axons and presynaptic terminals were inter-tangled in the EW nucleus, oculomotor nerve and CG, they could be distinguished from each other by the combination of XFPs using the Brainbow strategy. The individual presynaptic axons were extensively distributed with many branches and terminals in the CG at E8. At E10, some of the branches formed calyx-type endings but others did not. This is consistent with the results of a previous morphological study using electron microscopy [7]. The Brainbow color coding indicated that both calyx-type and bouton-like endings were formed from axon branches of the same neuron. As the frequency of branching of individual axons was reduced at E14, the majority of terminal arbors appeared to be removed by axon pruning [39]–[43]. We found debris of the axonal membrane frequently in the E14 ganglion. It is possible that these structures were phagosomes/lysosomes derived from axosomes engulfed by Schwann cells [44], [45], the main glial cells in the CG, because they usually contained multiple m-XFPs. In the developing and adult brain, microglia are suggested to reshape synapses and thus to be involved in the regulation of normal brain function such as learning and memory as well as in pathological reactions [46].

### Evaluation of Ca^2+^ Sensor Response

Neurotransmitter release from a presynaptic terminal is triggered by Ca^2+^ influx through voltage-dependent Ca^2+^ channels (VDCCs), classified into several subtypes and encoded by different genes [47]. In the calyx-type presynaptic terminals of chick CG, N-type Ca^2+^ channels (Ca_v_2.2) are mainly involved in the quantal release of acetylcholine, since a large fraction of the transmitter release as well as the presynaptic VDCC current are blocked by ω-conotoxin GVIA, a specific N-type VDCC blocker [18], [19], [48]. Only a small fraction of the VDCC current is resistant to ω-conotoxin GVIA, but insensitive to nifedipine, an L-type-blocking dyhydropyridine, or ω-agatoxin IVA, a P/Q-type-selective neurotoxin [19], [49], [50]. The VDCCs, particularly the N- and P/Q-type channels, are distributed in clusters in presynaptic terminals such as frog motor nerve terminal [51] and chick ciliary calyx [48], [52], [53]. Consequently, the intracellular Ca^2+^ concentration was elevated unevenly with hotspots in the presynaptic terminals during synaptic transmission in squid giant synapse [54] and frog motor nerve terminals [55]. A brief (<1 ms) elevation of local Ca^2+^ to 10–25 µM is sufficient to achieve the physiological transmission of the calyx of Held synapse [4]. Although a somewhat uneven elevation of Ca^2+^ was detectable in the present study, the change of fluorescence has no direct relationship to the focal Ca^2+^ concentration that regulates the transmitter release because of the relatively low *K*_d_ for Ca^2+^ (480 nM) of R-GECO1 [29]. Instead, it rather reflects the global Ca^2+^ elevation, which is dependent on the process of diffusion, sequestration and extrusion as well as Ca^2+^ inflow [20] and contributes to modifying the presynaptic machinery for a certain period [56]–[60]. Since a single postsynaptic ciliary cell usually receives a single presynaptic calyx-type terminal in the E14 CG, the relative change of the presynaptic Ca^2+^ dynamics could be directly related to the relative change of EPSP.

### Evaluation of ChR Expression

Optogenetics, which is based on genetically encoded molecules that couple light and neuronal function, enables one to manipulate a specific group of neurons in a complex of neural circuitry [61]. Various methods are now available for the targeted expression of exogenous molecules [25], [62]. In the present study, ChRFR gene was incorporated in the midbrain neurons in a non-specific manner using *in ovo* electroporation. However, only a subset of neurons in the EW nucleus innervates the CG through the oculomotor nerve to form calyx-type presynaptic terminals. A single visually-identified presynaptic terminal was thus optically stimulated under microscopic inspection without affecting the postsynaptic neurons or other presynaptic terminals. The direct irradiation of these terminals depolarized the membrane potential but hardly evoked action potentials. This is attributed to the relatively low input resistance of the presynaptic terminal (150±24 MΩ, n = 6) and the relatively small photocurrent (100±22 pA, n = 5). In the present study, this was overcome by partial blockade of K^+^ channels by 4-AP. However, the expression of ChRs and the efficiency of irradiation will need to be improved in the future.

We found that the [*ΔF*/*F*]_B_ of R-GECO1 was significantly slower in the transient when the presynaptic action potential was optically evoked in the ChRFR-expressing presynaptic terminal. The Ca^2+^ elevation that accompanied the activation of ChRFR would precede the invasion of the action potential in the presynaptic terminal because the [*ΔF*/*F*]_B_ was increased by the direct irradiation of the ChRFR-expressing terminal even in the presence of TTX. It is possible that the light-evoked depolarization facilitated Ca^2+^ inflow through presynaptic VDCCs (Figure S1). Alternatively, the Ca^2+^ would be translocated into the cytoplasm through ChRFR channels that are non-selective to cations [30]. The [*ΔF*/*F*]_B_ increased even in the absence of extracellular Ca^2+^ or cations. It was only slightly inhibited by xestrospondine C, negligibly by dantrolene, but significantly by thapsigargine. Therefore, the photo-activation of ChRFR appeared to facilitate the translocation of Ca^2+^ from intracellular Ca^2+^ stores via at least two routes, directly through its own channel in the calciosomal membrane or indirectly through binding of intracellular messengers to the calciosome receptors. This Ca^2+^ would bind to the high-affinity Ca^2+^-binding proteins in the cytoplasm in advance of the arrival of the action potential and thus modify the buffering and/or sequestration of the microdomain Ca^2+^ that flowed in through N-type VDCCs. Therefore, it is necessary to keep in mind that such background Ca^2+^ elevation could modify the biochemical milieu regulating the transmitter release [63]. The development of new ChRs, which are impermeable to Ca^2+^ would solve this problem by mimicking the direct current injection into presynaptic terminals.

### Conclusion

Recently, the calyx of Held in the medial nucleus of the trapezoid body of rodents has been used as a model glutamatergic excitatory synapse and has been extensively studied to reveal the physiological basis of presynaptic mechanisms [64], [65]. Similar to the calyx of Held synapse, the calyx-type presynaptic terminal of CG is relatively large and therefore accessible to physiological recording methods such as the patch clamp. As it innervates the postsynaptic ciliary neuron in a one-to-one manner at E14, its physiology corresponds to the morphology and electrophysiological responses of its postsynaptic cell. Further progress can be expected if the presynaptic mechanisms of these large terminals can be studied using genetic modifications [66]. Here, we removed the obstacles that for a decade hindered molecular studies of the calyx-type presynaptic terminal and provided an experimental platform that will accelerate investigations of the presynaptic terminal in terms of morphology, physiology and development.

## Materials and Methods

### Ethics Statement

All animal experiments were approved by the Tohoku University Committee for Animal Experiments (Approval No. 2012LsA-3) and were carried out in accordance with the Guidelines for Animal Experiments and Related Activities of Tohoku University as well as the guiding principles of the Physiological Society of Japan and the National institutes of health (NIH), USA. In the present study, chick embryos were used for experiments at stages no later than E14.

### Chick Embryo and Tissue Preparation

White Leghorn embryonated chick eggs (Koiwai Farm Products, Ltd., Shizukuishi, Japan) were incubated at 38°C in a humid atmosphere until they reached the appropriate stages [67]. At each stage (E8: stage 33–34; E10: stage 35–36; E14: stage 39–40), the embryos were decapitated, the brain stem, oculomotor nerve and CG were excised and used for the physiological or histological experiments.

The isolated brainstem was further fixed with 4% paraformaldehyde (PFA) in PBS (pH 7.8–8.0) for 1.5 h at 4°C and then sliced into 200 µm-thick sagittal sections in PBS using a microtome (CM 3050S, Leica) and used for the microscopic examination.

### Plasmid Vectors

pCAGGS-EGFP was made as described previously [68]. pCAGGS-R-GECO1 and pCAGGS-Brainbow1.1M: the coding region of each pCMV-R-GECO1 (plasmid 32444, Addgene, Cambridge MA, USA) and pCMV-Brainbow1.1M (plasmid 18722, Addgene) was subcloned into pCAGGS. pCAGGS-mCherry-NCre: the NCre construct, a kind gift from Dr. Ishihara (Tohoku University, Sendai, Japan), was inserted into pmCherry-N1. The coding region of the product was subcloned into pCAGGS. pCAGGS-ChRFR-EGFP: the ChRFR construct [30] was inserted into pEGFP-N1. The coding region of the product was subcloned into pCAGGS.

### *In ovo* Electroporation

Plasmid vectors, pCAGGS-EGFP, pCAGGS-Brainbow1.1M, pCAGGS-mCherry-NCre, pCAGGS-R-GECO1 and pCAGGS-ChRFR-EGFP were used for the *in ovo* electroporation as described previously [26], [27]. Briefly, the plasmid vectors (0.2–0.3 µg/µL) were injected with fastgreen into the midbrain ventricular space of E2 chick embryos (stage 14–15), a pair of parallel electrodes was placed on the embryonic brain so as to sandwich the midbrain region, and a rectangular pulse (25 V, 50 ms) was charged four times at an interval of 950 ms (CUY21EDIT, BEX company, Tokyo, Japan).

### Electrophysiology

#### EPSC recordings

The electrophysiological recording techniques were as described previously [69], [70]. Briefly, a whole ganglion was mounted in a superfusion chamber (ca. 1 ml); the oculomotor nerve was drawn to the stimulating electrode by suction, and the collagenous envelope was enzymatically removed by focally applying a mixture of collagenase (type II, 3000 U/ml; Sigma-Aldrich, St. Louis MO, USA) and thermolysin (100 U/ml, Sigma-Aldrich) through a glass pipette (tip diameter, 40–50 µm). The ganglion was superfused with standard Tyrode solution containing 138 mM NaCl, 3 mM KCl, 10 mM HEPES, 4 mM NaOH, 5 mM CaCl_2_, 1 mM MgCl_2_ and 11 mM glucose, adjusted to pH 7.4 by 1 N HCl. All experiments were performed at room temperature (25°C). A conventional whole-cell patch-clamp recording was made from a postsynaptic ciliary neuron using an EPC-7 plus patch-clamp amplifier (HEKA Elektronik, Lambrecht, Germany). Patch pipettes (5–7 MΩ) were filled with an internal solution containing 120 mM CsOH, 120 mM gluconic acid, 10 mM CsCl, 10 mM EGTA, 10 mM HEPES, 1 mM MgCl_2_, 3 mM MgATP, 0.1 mM leupeptin and 5 mM QX-314, adjusted to pH 7.2 by CsOH. The series resistance was usually ∼10 MΩ throughout the experiment. EPSC was measured at a holding potential of −60 mV. To ensure the stable recording of EPSC, the capacitative transient was minimized by the electrical circuitry, and the series resistance was compensated for by 50–70%. The whole-cell currents were low-pass filtered at 1 kHz, digitized at 10 kHz (Digidata 1200 series interface, Molecular Devices, LLC, Sunnyvale CA, USA) and stored in a computer using software (pCLAMP 8.2, Molecular Devices, LLC).

#### Presynaptic recording

The collagenous envelope of the CG was enzymatically removed in Tyrode solution containing a mixture of collagenase (type II, 300 U/ml; Sigma-Aldrich, St. Louis, MO, USA) and thermolysin (10 U/ml, Sigma-Aldrich) for 30 min at 37°C [70]. The ganglion was adsorbed to a membrane filter and used for the experiments. The calyx-type nerve terminal was identified by the presence of EGFP fluorescence. A conventional whole-cell patch-clamp recording was made from a calyx-type presynaptic terminal as described previously [19] using an EPC-8 patch-clamp amplifier (HEKA Elektronik). The pipette solution for the presynaptic recording contained 125 mM K-gluconate, 10 mM KCl, 0.2 mM EGTA, 10 mM HEPES, 1 mM MgCl_2_, 0.3 mM Na_2_GTP, 10 mM Na_2_-phosphocreatine and 0.1 mM leupeptin, adjusted to pH 7.2 by KOH. The signals were acquired using software (Clampex 10.2, Molecular Devices, LLC).

### Optical Stimulation

A fiber-coupled 451-nm laser source (Optohub, Saitama, Japan) was used for the optical stimulation. The free end of the optic fiber (core diameter; 50 µm, Doric Lenses, Quebec City, Canada) was placed closed to the calyx-type presynaptic terminal. The timing of the stimulation was controlled by a stimulator (SEN-7203, Nihon Kohden, Tokyo, Japan) and software (Clampex 8.2, Molecular Devices, LLC) and triggered by a laser-confocal microscopy system. The maximal power of the laser light, 18.7 µW, was directly measured at the free end of the optic fiber by a thermopile (MIR-100Q, Mitsubishi Oil Chemicals, Tokyo, Japan).

### Image Acquisition and Analysis

Morphological imaging of each fluorescence in Fig. 1 and 2 was carried out using a conventional laser-scanning confocal microscopy system (LSM710, Carl Zeiss, Oberkochen, Germany) equipped with 20× dry objectives (0.5 NA) and 40× oil-immersion objectives (1.3 NA) in the following optical combinations: a 488-nm argon laser and 493–576-nm bandpass slider for EGFP, a 440-nm photodiode laser and 454–522-nm bandpass slider for CFP, a 514-nm argon laser and 523–581-nm bandpass slider for YFP and a 561-nm DPSS laser and a 581–727-nm bandpass slider for RFP. The CFP, YFP and RFP images in Brainbow were acquired sequentially in each XY plane. Z-stack images were converted to RGB tiffs with ImageJ software (http://rsb.info.nih.gov/ij/) and maximally projected three-dimensionally using FluoRender software (http://www.fluorender.com, [71]).

Images of R-GECO1 fluorescence were acquired using a high-speed laser-scanning confocal microscopy system (A1R, Nikon, Tokyo, Japan) equipped with 16× water-immersion objectives (0.8 NA), a 561-nm DPSS laser, a 405/488/561/640-nm dichroic mirror and a 580±23-nm bandpass filter. The images were sampled at 7.62 fps (galvano scan; 256×256 pixels) for Fig. 3 B–D or 133.11 fps (resonant scan; 512×128 pixels) for other experiments. Each exact sampling speed was calculated on the basis of the periodical signals of Clampex software. The fluorescence in the calyx was captured as a whole with a 25.65 µm optic slice (17.4 airy units) unless otherwise noted. The calyx-type presynaptic terminal was identified visually by the co-expression of EGFP fluorescence. The oculomotor nerve was drawn to the stimulating electrode by suction and stimulated electrically (200 µs pulse). Some presynaptic terminals were optically stimulated during the imaging.

Image analysis was performed with ImageJ software. After median-filtered noise reduction at 5 pixels, a region of interest (ROI) was set to cover a single calyx-type terminal under visual identification. The fluorescence intensity in each ROI was sampled as the time series of digits and analyzed with Excel software (Microsoft Japan, Tokyo, Japan). The fluorescence change was defined as *ΔF*/*F* = (*F*_t_−*F*_0_)/*F*_0_, where *F*_t_ is the fluorescence intensity at time *t*, and *F*_0_ is the average baseline fluorescence 1 s before the stimulation. The maximal *ΔF*/*F* within 1 s after the stimulation was used as the peak magnitude of the Ca^2+^ transient. The rising phases of *ΔF*/*F* were fitted between 25 and 130 ms after the stimulation to a single exponential function with the time constant, τ_R_, using software (Clampfit 10.2, Molecular Devices, LLC). Similarly, the decaying phase of *ΔF*/*F* was fitted between 80 and 0% of peak amplitude with a time constant, τ_D_.

The R-GECO1 imaging was performed under superfusion with standard Tyrode solution or with cation-free isotonic solution of the following formula; 290 mM mannitol, 1 mM EGTA, 10 mM HEPES, 6 mM TEAOH, 5 mM MgCl_2_ and 11 mM glucose (adjusted to pH 7.4 by HCl).

### Reagents

Regents used in this study and their sources were as follows: 4-aminopyridine (4-AP, 1 mM; Nacalai tesque, Kyoto, Japan); tetrodotoxin (TTX, 1 µM; Tocris Bioscience, Bristol, UK); QX-314 (Tocris Bioscience); xestospongin C (Enzo Life Sciences, Farmingdale, NY); dantrolene (Sigma-Aldrich); thapsigargin (Tocris Bioscience). Xestospongin C, dantrolene and thapsigargin were dissolved in DMSO and diluted in the recording chamber (1 ml) while stopping superfusion. The concentration of DMSO was <0.5%.

### Statistics

All data in the text are presented as the mean ± SEM (number of experiments). Wilcoxon signed rank test was used for statistical analysis of paired samples unless otherwise noted.

## Supporting Information

Figure S1 **Contribution of voltage-dependent Ca^2+^ channels to the optogenetic Ca^2+^ mobilization. A**, Typical [*ΔF/F*]_B_ changes in the TTX-treated presynaptic terminal: the response to a single 20 ms laser pulse (blue) and that in the presence of 10 µM ω-conotoxin GVIA (CgTx) (red). Each trace is an average of five consecutive records. **B**, Summary of peak [*ΔF/F*]_B_ changes (mean ± SEM) in the presence of TTX; with (red) and without CgTx (blue). **C**, Sample [*ΔF/F*]_B_ responses of the same presynaptic terminal as shown in **A**, but those to a train of laser pulses (10 Hz, 1 s) with (red) and without CgTx (blue). Each trace is an average of five consecutive records. **D**, Summary of peak [*ΔF/F*]_B_ changes (mean ± SEM) in the presence of TTX; with (red) and without CgTx (blue). *P<0.05, Wilcoxon signed rank test (n = 6).
(PDF)

Video S1 Three-dimensional reconstruction of the confocal images of an EGFP-expressing calyx-type presynaptic terminal in the CG. Note that EGFP was expressed neither in the postsynaptic cell nor in the Schwann cells.
(AVI)

Video S2 Three-dimensional reconstruction of the confocal images of a CG at E8 (the same sample shown in Fig. 2F). Each presynaptic axon was colored according to the combination of expressed CFP, YFP and RFP under Brainbow strategy.
(AVI)

Video S3 Three-dimensional reconstruction of confocal images of a CG at E10 (the same sample shown in Fig. 2H). Each presynaptic axon was colored according to the combination of expressed CFP, YFP and RFP under Brainbow strategy.
(AVI)

Video S4 Three-dimensional reconstruction of confocal images of a CG at E14 (the same sample shown in Fig. 2J). Each presynaptic axon was colored according to the combination of expressed CFP, YFP and RFP under Brainbow strategy.
(AVI)

Video S5 R-GECO1 fluorescence in a calyx-type presynaptic terminal in response to a single electrical stimulation of the oculomotor nerve. A series of pseudocolor-rated images in real time.
(AVI)
