# Supplementary figures and images for: Optogenetic Probing and Manipulation of the Calyx-Type Presynaptic Terminal in the Embryonic Chick Ciliary Ganglion
Source: PLoS One. 2013 Mar 21;8(3):e59179. doi: 10.1371/journal.pone.0059179 (PMC3605445; doi:10.1371/journal.pone.0059179)

Figure S1

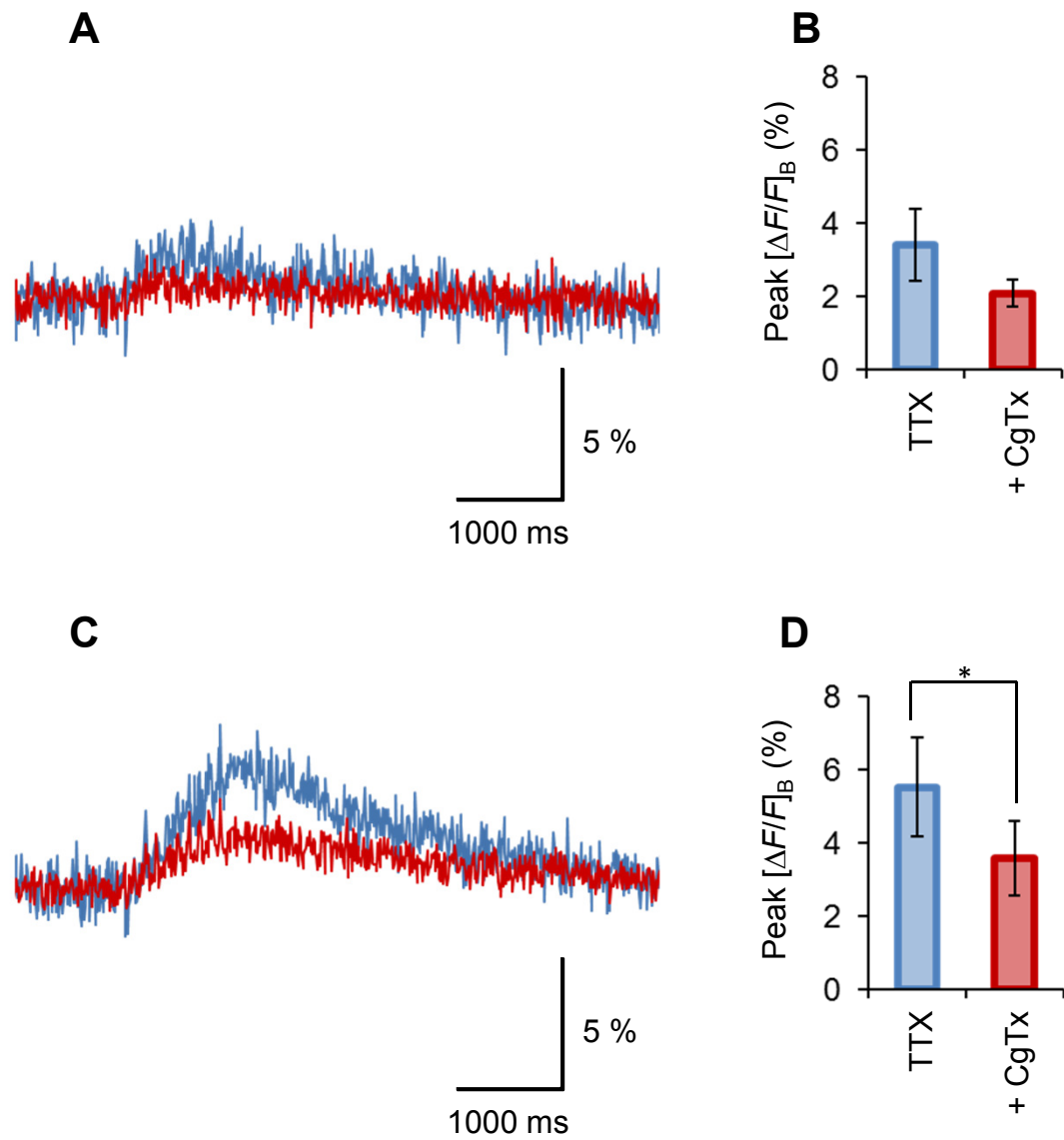

Supplement: Figure S1 — Contribution of voltage-dependent Ca2+ channels to the optogenetic Ca2+ mobilization. A, Typical [ΔF/F]B changes in the TTX-treated presynaptic terminal: the response to a single 20 ms laser pulse (blue) and that in the presence of 10 µM ω-conotoxin GVIA (CgTx) (red). Each trace is an average of five consecutive records. B, Summary of peak [ΔF/F]B changes (mean ± SEM) in the presence of TTX; with (red) and without CgTx (blue). C, Sample [ΔF/F]B responses of the same presynaptic terminal as shown in A, but those to a train of laser pulses (10 Hz, 1 s) with (red) and without CgTx (blue). Each trace is an average of five consecutive records. D, Summary of peak [ΔF/F]B changes (mean ± SEM) in the presence of TTX; with (red) and without CgTx (blue). *P<0.05, Wilcoxon signed rank test (n = 6). (PDF) [file pone.0059179.s001.pdf]
